# Cationic Liposomes Carrying HPV16 E6-siRNA Inhibit the Proliferation, Migration, and Invasion of Cervical Cancer Cells

**DOI:** 10.3390/pharmaceutics16070880

**Published:** 2024-06-29

**Authors:** Luz Victoria Sánchez-Meza, Ciresthel Bello-Rios, Josimar O. Eloy, Yazmín Gómez-Gómez, Marco Antonio Leyva-Vázquez, Raquel Petrilli, María Josefa Bernad-Bernad, Alfredo Lagunas-Martínez, Luis Alberto Medina, Janeth Serrano-Bello, Jorge Organista-Nava, Berenice Illades-Aguiar

**Affiliations:** 1 Facultad de Ciencias Químico Biológicas, Universidad Autónoma de Guerrero, Av. Lázaro Cárdenas S/N, Ciudad Universitaria, Chilpancingo 39090, Guerrero, Mexico; luzsanchez@uagro.mx (L.V.S.-M.); ciresthelbello@uagro.mx (C.B.-R.); yazmingomez@uagro.mx (Y.G.-G.); leyvamarco13@gmail.com (M.A.L.-V.); 2 Department of Pharmacy, Dentistry and Nursing, Faculty of Pharmacy, Federal University of Ceará, 1210 Pastor Samuel Munguba Street, Fortaleza 60430-160, CE, Brazil; josimar.eloy@gmail.com; 3 Institute of Health Sciences, University of International Integration of the Afro-Brazilian Lusophony, Redenção 62790-000, CE, Brazil; petrilliraquel@unilab.edu.br; 4 Facultad de Química, Universidad Nacional Autónoma de Mexico, Ciudad de Mexico 04510, Mexico; bernadf@gmail.com; 5 Centro de Investigación sobre Enfermedades Infecciosas, Instituto Nacional de Salud Pública, Cuernavaca 62100, Morelos, Mexico; alagunas@insp.mx; 6 Instituto de Física, Universidad Nacional Autónoma de Mexico, Ciudad de Mexico 04510, Mexico; medina.lamv@gmail.com; 7 Unidad de Investigación Biomédica en Cáncer INCan/UNAM, Instituto Nacional de Cancerología, Actualmente Hospital Ángeles Puebla, Ciudad de Mexico 14080, Mexico; 8 Facultad de Odontología, Universidad Nacional Autónoma de Mexico, Ciudad de Mexico 04360, Mexico; janserbello@fo.odonto.unam.mx

**Keywords:** cervical cancer, E6 oncoprotein, HPV16, E6 siRNAs, lipoplexes, liposomes

## Abstract

The E6 and E7 oncoproteins of high-risk types of human papillomavirus (HR-HPV) are crucial for the development of cervical cancer (CC). Small interfering RNAs (siRNAs) are explored as novel therapies that silence these oncogenes, but their clinical use is hampered by inefficient delivery systems. Modification (pegylation) with polyethylene glycol (PEG) of liposomal siRNA complexes (siRNA lipoplexes) may improve systemic stability. We studied the effect of siRNA targeting HPV16 E6, delivered via cationic liposomes (lipoplexes), on cellular processes in a cervical carcinoma cell line (CaSki) and its potential therapeutic use. Lipoplexes-PEG-HPV16 E6, composed of DOTAP, Chol, DOPE, and DSPE-PEG2000 were prepared. The results showed that pegylation (5% DSPE-PEG2000) provided stable siRNA protection, with a particle size of 86.42 ± 3.19 nm and a complexation efficiency of over 80%; the siRNA remained stable for 30 days. These lipoplexes significantly reduced HPV16 E6 protein levels and restored p53 protein expression, inhibiting carcinogenic processes such as proliferation by 25.74%, migration (95.7%), and cell invasion (97.8%) at concentrations of 20 nM, 200 nM, and 80 nM, respectively. In conclusion, cationic lipoplexes-PEG-HPV16 E6 show promise as siRNA carriers for silencing HPV16 E6 in CC.

## 1. Introduction

Cervical cancer (CC) is the fourth most common cancer in women, and more than 85% of the CC deaths recorded annually occur in less developed regions, ranking second in incidence and mortality behind breast cancer in these areas [1,2]. Human papillomavirus (HPV) has been identified as the critical etiological agent for the development of CC. HPVs are classified into high-risk (HR-HPV), medium-risk (MR-HPV), and low-risk (LR-HPV) types, according to their ability to develop cancer [3]. Among the high-risk HPV genotypes, HPV16 and 18 are primarily associated with CC [4]. Epidemiological and experimental studies indicate that infection with HR-HPV is necessary but insufficient to induce CC; such infection is required to cause the genetic and epigenetic changes that alter carcinogenesis-related cellular processes [5]. Although chemotherapy and other treatments used for CC are effective, they are highly invasive and cytotoxic and have low bioavailability, which limits their use in cancer treatment [6].

Nucleic acid-based therapy has emerged as a highly promising and innovative approach for the treatment of a wide range of acquired and inherited diseases [7]. This innovative strategy involves the transfer of therapeutic genes, whether DNA or RNA, into target tissues to either facilitate gene expression or suppress specific genes. One prominent tool utilized in this type of therapy is small interfering RNAs (siRNAs). The siRNAs consist of short double-stranded RNA molecules that effectively repress the expression of target genes through the precise degradation of complementary mRNA sequences [8]. Although siRNA-mediated target gene degradation is a powerful method, it faces certain intrinsic limitations, including its short half-life and rapid clearance by the mononuclear phagocytic system (MPS), which eliminates it through opsonization and phagocytosis processes as part of the immune response against circulating foreign agents. Furthermore, the presence of biological barriers further complicates the delivery of siRNA to target tissues and low cellular uptake due to repulsion by the negatively charged cell membrane [9,10,11]. Hence, the development of suitable vectors for the efficient and stable delivery of gene therapy is of paramount importance [12]. Developing nanoscale vectors to deliver nucleic acids as a treatment for various diseases is a crucial aspect of this endeavor. Consequently, vectors have been divided into viral and non-viral systems. Viral vectors are efficient in transfection [7,13], but they pose a risk to the host due to the immunogenicity of their viral proteins, the potential for oncogenesis due to chromosomal integration, and the generation of infectious viruses due to recombination [14]. In contrast, non-viral vectors offer advantages, such as the ease of modifying their surface to target specific tissues, their lack of immunogenicity, their relative safety, and the ease of large-scale production. For these reasons, non-viral vectors are considered promising alternative methods for gene therapy [15,16]. Nanoparticles (NPs) have emerged as up-and-coming tools in nucleic acid-based therapy due to their advantageous physicochemical properties that can be tailored to enhance their performance [17]. Among the various types of nanoparticles used in nucleic acid-based therapy, liposomes and cationic polymers have received significant attention due to their versatility and efficacy [18,19,20]: they can easily form complexes (genetics and liposomes) with DNA or RNA because of the negative charges of the phosphate groups of nucleic acids. The loading of liposomes offers numerous advantages in nucleic acid-based therapy, including targeted delivery, enhanced cellular internalization, improved bioavailability, reduced toxicity, and potential for co-delivery, among others [12,21].

The small interfering RNAs (siRNAs) are an innovative research tool with great potential in cancer treatment. This innovative technique has shown promising results in reducing the expression of oncogenes, such as E6 and E7 [22,23,24,25,26], because the interaction of E6 with several molecular targets that may support its oncogenic activity, such as the interaction with Bcl-2 (Bak) that is associated with an increase in its proteolytic turnover rate through the E6AP proteasome pathway or with E6 interaction with p300/CBP, E6BP/ERC-55, MCM7, c-Myc, and paxillin that is strongly associated with cell transformation and apoptosis is well documented [27,28,29]. Notably, targeting the entire E6/E7 transcript with a single siRNA can silence the expression of both oncogenes [23], inducing apoptosis, cell growth arrest, and cell senescence, due to the reactivation of tumor suppressor pathways such as p53 and Rb [30,31,32]. Jonson et al. 2008 demonstrated that the use of siRNAs targeting the major HPV oncogenes in HPV-induced tumors can successfully inhibit tumor growth. This groundbreaking study highlights the ability of siRNA to silence both oncogenes simultaneously and emphasizes the potential of siRNA-based treatments for various cancer types [33]. However, it is necessary to mention that the E6 and E7 oncogenes in CC-positive cells generate a state of genomic instability that leads them to express variants and isoforms [34,35] inducing an increase in the development of secondary tumors [36]. Due to this instability caused by the isoforms and variants of the E6 and E7 oncogenes, genomic heterogeneity has been observed among patients with CC. Therefore, the design of therapies efficiently targeting these proteins is necessary to develop therapeutic alternatives based on siRNAs to inhibit the expression of these target genes. In this study, we designed an siRNA targeting HPV16 E6 isoforms, coupled to cationic liposomes. Specifically, we incorporated an E6 siRNA into a lipoplex, which increased the stability of the system and the efficacy of delivery to the CaSki cell line, allowing us to reduce the expression of the HPV16 E6 oncoprotein.

## 2. Materials and Methods

### 2.1. Materials

The 1,2-dioleoyl-3-trimethylammonium-propane (DOTAP), 1,2-dioleoyl-sn-glycero-3-phosphoethanolamine (DOPE), cholesterol, and 1,2-distearoyl-sn-glycero-3-phosphoethanolamine-N-[methoxy(polyethylene glycol)-2000] (DSPE-PEG2000) were purchased from Avanti Polar Lipids, Inc. (Alabaster, AL, USA). A Quant-iTTM RiboGreen^®^ RNA Reagent and Kit (Invitrogen, Life Technologies, Eugene, OR, USA), RNase A PureLink™ (Thermo Fisher, Life Technologies, Carlsbad, CA, USA), and Recombinant RNasin^®^ Ribonuclease Inhibitor (Promega, Madison, WI, USA) were used. CaSki cells (derived from human cervical carcinoma) were obtained from the American Type Culture Collection (ATCC) (Manassas, VA, USA). Fetal bovine serum (FBS) (Gibco, Life Technologies, Carlsbad, CA, USA), MEM Non-Essential Amino Acid Solution (Minimum Essential Medium) (Gibco, Life Technologies), 100 U/mL penicillin, 100 mg/mL streptomycin (Gibco, Life Technologies), and Trypsin-EDTA (ethylene diamine tetraacetic acid) were purchased from Gibco (Gibco, Life Technologies). The siRNAs against E6 were obtained from Integrated DNA Technologies (IDT, Integrated DNA Technologies, Cambridge MA, USA), and MTT (Cell Proliferation Kit I) was obtained from Roche (Roche, Mannheim, Germany).

### 2.2. Design of the E6 siRNAs

A search for the E6 sequence of HPV16 (UniProt, NCBI) was performed. Subsequently, an in silico analysis was used for the multiple alignment of nucleotide sequences (European Nucleotide Archive < EMBL-EBI) and amino acids (UniProt) to identify conserved sequences between these HR-HPVs. The following siRNA sequences were obtained: siRNA E6 sense chain 5′-AAAAGAGAACUGCAAUGUUUCAGGA-3′ and antisense chain 5′-UCCUGAAACAUUGCAGUUCUCUUUUGG-3′.

### 2.3. Preparation of Liposomes

Following the methodology described by Hattori in 2019, we prepared liposomes using a mixture of DOTAP, cholesterol, and DOPE at a molar concentration of 2:1:1 [37] via the lipid film rehydration method [38]. The lipids were dissolved in chloroform (CHCl3), which was subsequently removed using a rotary evaporator at 40 °C for 30 min at 100 rpm. The thin lipid film was hydrated with RNase-free water for 40 min in a rotary evaporator at 40 °C and 100 rpm. Next, a sonicator was used to reduce the particle size (amplitude, 30%; 20 s on and 10 s off for 10 min) [39].

### 2.4. Preparation of Lipoplexes

HPV16 E6-siRNA was mixed with cationic liposomes at a concentration of 100 nM diluted in RNase-free water and incubated at room temperature for 30 min [39].

### 2.5. Preparation of Pegylated Liposomes

The liposomes were pegylated by adding DSPE-PEG2000 at a molar ratio of 5% of the total lipids [40]. DSPE-PEG2000 was added to the lipid mixture in a rotary evaporator with CHCl3. The resulting mixture was then subjected to sonication as indicated above.

### 2.6. Particle Size and Zeta Potential Measurements

The dynamic laser light scattering method was used to determine the mean size of the lipoplexes and the polydispersity index (PDI). The viscosity and refractive index of pure water were used. The charge density of the lipoplexes was evaluated by examining their Z-potential (mV), and both measurements were performed using a Zen 3600 zeta-sizer (Malvern Instruments, Malvern, UK). The concentration of each formulation was measured ten times (*n* = 10) in triplicate.

### 2.7. Evaluation of Particle Morphology

Transmission electron microscopy (TEM) (JEM-2010, Jeol Co., Tokyo, Japan) with an LaB6 electron beam at 200 kV was used to evaluate morphology and size. Briefly, a drop of the sample was placed on a copper grid coated with 400-mesh carbon film and allowed to dry for 5 min. Then, the sample was stained with a 2% uranyl acetate solution and allowed to dry before being viewed in the microscope. Sizing was performed with ImageJ bundled with 64-bit Java 1.6.0-24 software (NIH, Bethesda, MD, USA) by measuring the diameter of 30–50 images of liposomes and calculating a mean diameter.

### 2.8. Characterization of Lipoplexes and Encapsulation Efficiency

#### 2.8.1. Gel Retardation Assay

The encapsulation of the HPV16 E6-siRNA in the pegylated liposomes (lipoplexes-PEG-HPV16 E6) was determined by electrophoresing the sample on a 4.5% agarose gel [41]. Lipoplexes-PEG-HPV16 E6 were prepared in RNase-free water and loaded with the siRNA at a concentration of 1 µM. Samples of liposomes and lipoplexes-PEG-HPV16 E6 were loaded into an agarose gel and separated using 1 × TAE as the running buffer at 80 mV for 35 min. The gel was stained with ethidium bromide and visualized under UV light using a Gel Doc XR molecular imaging system (Bio-Rad, Hercules, CA, USA).

#### 2.8.2. Quant-iTTM RiboGreen^®^ RNA Assay

Following the manufacturer’s protocol, the coupling efficiencies were measured using the RiboGreen^®^ Quant-iTTM RNA assay (Invitrogen, Life Technologies, Portland, OR, USA). The lipoplexes-PEG-HPV16 E6 and liposomes were incubated for 1 h with Triton buffer (10% Triton X-100, Sigma Aldrich, Saint Louis, MO, USA). Subsequently, RiboGreen reagent was added to each incubated sample, and the fluorescence signal was recorded using a fluorescence reader (GloMaxR-Multi Jr.). The amount of noncoupled siRNA-HPV16 E6 (Fu) was determined with TE buffer-incubated liposomes (Fu), and the total siRNA amount (Ft) was calculated from the Triton buffer-lysed samples. The encapsulation efficiency (%) = (Ft − Fu)/Ft × 100.

### 2.9. Stability Study

#### 2.9.1. siRNA Integrity

The integrity of the siRNA pair was analyzed via agarose gel electrophoresis. Triton X-100 (10% *w*/*v*) was used as a detergent to disrupt the liposomes-HPV16 E6 and extract siRNA from the complex. The suspension was vortexed and then incubated for 1 h at room temperature. The gel retardation assay was performed under the conditions described above.

#### 2.9.2. Loss of the siRNA

The lipoplexes-PEG-HPV16 E6 were stored at 4 °C for 30 days. The loss of the siRNA was quantified using the RiboGreen^®^ assay (mentioned above) on the first day and 30 days after preparation.

#### 2.9.3. siRNA Protection from RNase

Lipoplexes-PEG-HPV16 E6 were incubated in RNase A (0.05 mg/mL) for 2 h at 37 °C. Then, RNaseOUTTM (40 IU/mL) and Triton X-100 (10% *w*/*v*) were added, and the suspensions were mixed and incubated at room temperature for one h. The free siRNA was visualized via a gel retardation assay.

### 2.10. Cultured Cell Lines

The HaCaT cell line (negative for HPV) was maintained in Dulbecco’s modified Eagle’s medium (Gibco, Life Technologies). In contrast, the CaSki (positive for HPV16) cell line was maintained in modified Eagle’s medium (Gibco, Life Technologies) supplemented with 10% fetal bovine serum (Gibco, Life Technologies), 100 U/mL penicillin, and 100 μg/mL streptomycin (Gibco, Life Technologies) and incubated at 37 °C in a humidified atmosphere with 5% CO_2_.

### 2.11. Transfection with Lipoplexes

CaSki and HaCaT cells were treated with lipoplexes-PEG-HPV16 E6 at siRNA concentrations of 20 nM, 40 nM, 80 nM, 160 nM, and 200 nM for the different assays performed, and the results were evaluated at different time points (0, 24, and 48 h). The cells were cultured with MEM in the absence of lipoplexes-PEG-HPV16 E6 as a control; as a second control, the cells were cultured with vehicle (H_2_O) and plated at the volume of the highest concentration of siRNA applied to the cells.

### 2.12. Western Blot

Forty-eight hours after transfection, the cells were harvested and lysed. Protein was extracted with RIPA lysis and extraction buffer (Thermo Fisher Scientific, Inc., Waltham, MA, USA). The Bradford method (Bio-Rad, Hercules, CA, USA) was used to measure the protein concentrations. Samples containing 30 µg of protein were boiled in SDS-containing sample buffer, separated by SDS polyacrylamide gel electrophoresis (SDS-PAGE), and transferred to nitrocellulose membranes (Bio-Rad, Hercules, CA, USA). The membranes were blocked with 5% nonfat milk in TBS containing 0.05% Tween-20 for 2 h and probed with the appropriate antibody dilution. HPV16 E6 (MA146057, Thermo Fisher Scientific, Inc., USA) and GAPDH antibodies (sc-47724, Santa Cruz Biotechnology, USA) were used at a dilution of 1:1000, and the p53 antibody (sc-126, Santa Cruz Biotechnology, Dallas, TX, USA) was used at a dilution of 1:500. The HRP-conjugated anti-mouse secondary antibody (sc-2005; Santa Cruz Biotechnology, USA) was subsequently applied at a dilution of 1:10,000. The proteins were visualized through an enhanced chemiluminescence reaction using Pierce™ ECL Western blotting Substrate (Thermo Scientific, Pierce Protein Research Products, Rockford, IL, USA), and Western blot images were captured with an InvitrogenTM iBright CL750 Imaging System (Life Technologies, Marsiling Industrial Estate Road, Singapore) and quantified using ImageJ software (version 1.41, National Institutes of Health, Bethesda, MD, USA).

### 2.13. Cell Viability and Proliferation Assays

Treated CaSki and HaCaT cells were seeded in 96-well plates at a density of 6 × 10^3^ cells per well, incubated for 24 h, and synchronized for 24 h. Then, different treatments were administered for 48 h. Control cells were treated in the same manner but without the addition of liposomes. The cells were treated with Cell Proliferation Kit I (MTT) reagent (Roche, Mannheim, Germany) as indicated by the manufacturer, and the absorbance was measured at 570 nm with a Thermo Scientific Multiskan FC (Thermo Labsystems, Milford, CT, USA). Cytotoxicity was reported as the mean percentage of viable cells after treatment relative to that of untreated cells. All the conditions were tested in triplicate.

### 2.14. Cell Migration Assay

CaSki cells were seeded in 6-well plates and cultured until they reached the desired confluence. Subsequently, the cells were serum-starved for 4 h; after the first 2 h of incubation, Ara C (1X) (Sigma Aldrich, Saint Louis, MO, USA) was added and incubated for 2 h. Then, the cells were scratched using a sterile 200 μL micropipette tip. The cells detached by scratching were removed by washing with 1X PBS. Different concentrations of lipoplexes-PEG-HPV16 E6 were then added to the supplemented MEM. Images of the cells were captured at 0 h and 48 h after wounding using an inverted microscope with an attached camera. At each time point, the width of the scratch area was averaged at five points to enable better measurement of migration of cells from the monolayer into the wound area over time. Each condition was tested in triplicate. Migration was evaluated using ImageJ software.

### 2.15. Matrigel Invasion Assay

Upon reaching the desired confluence, the CaSki cells were serum-starved for 4 h, harvested, and suspended in a basal medium. Matrigel (8 μg/mL) (Sigma Aldrich, St. Louis, MO, USA) was added to each Transwell chamber (Corning, St. Lowell, MA, USA). The chambers were incubated at 37 °C for 2 h. Subsequently, 1 × 10^5^ cells were seeded onto the upper part of the Transwell chamber. Different treatments were introduced into the upper part of the chamber with a medium supplemented with 0.1% fetal bovine serum (FBS). The lower chamber was filled with a 5% supplemented medium. The chamber was incubated in an atmosphere of 5% CO_2_ at 37 °C for 48 h. The cells were fixed with cold methanol, and the noninvading cells were removed from the upper side of the membrane using water and a swab. Then, the cells were stained with 0.1% crystal violet and washed three times with water to remove excess dye. Photographs were captured, and the invading cells were counted. The invasion was evaluated using ImageJ software.

### 2.16. Statistical Analysis

The data are presented as the means ± standard deviations (SDs). Statistical analyses were performed using one-way analysis of variance (ANOVA) with Dunnett’s multiple comparison test, Tukey’s test, the Bonferroni correction, or the test indicated for each analysis, and Prism 9.0.1 software was used (GraphPad Software, San Diego, CA, USA). A value of * *p* < 0.05 indicated statistical significance.

## 3. Results and Discussion

### 3.1. Comprehensive Characterization of Liposomes and Lipoplexes: Elucidating the Key Features for Effective Drug Delivery

Designing particles with precise physicochemical characteristics is crucial for the development of lipid-based nanoparticles with heightened resistance to aggregation in the bloodstream [42]. Optimizing the particle size, polydispersity index (PDI), and zeta potential (Z-potential) can ensure exceptional biocompatibility and viability in the specific systems under study [43]. The PEG_2000_ polymer has increased in importance as a degradation protection system, stability enhancer, and circulation time extender for siRNA-targeted liposomes [37,44].

Cationic liposomes composed of DOTAP, DOPE, and cholesterol were prepared with and without DSPE-PEG2000. It has been demonstrated in the literature that formulations based on DOTAP, Chol, DOPE, and the addition of the polymer DSPE-PEG2000 have the characteristic of improving siRNA encapsulation, stability, and cellular internalization, compared to other formulations [42,45]. The lipoplexes-PEG-HPV16 E6 exhibited a narrow particle size distribution ranging from approximately 88 to 95 nm, indicating a uniform size. In addition, they exhibited a PDI ≤ 0.3, highlighting the ability of the liposomes to encapsulate the therapeutic payload efficiently. Furthermore, lipoplexes-PEG-HPV16 E6 showed a positive Z-potential between 47 and 50 mV. The incorporation of E6 siRNA into both pegylated and non-pegylated liposomes showed no differences in the physicochemical properties between the two types of liposomes (Table 1). Similar data were reported using the same molar ratio of 2:1:1, presenting values with particle size ranging from 104.9 to 2014.7 nm in diameter and a Z-potential of 47.6 to 42.1 mV, in liposomes without and with siRNA, respectively [37], in formulations with and without DSPE-PEG2000, values were observed with particle sizes ranging between 92 and 145 nm, with PDI greater than 0.28 and a Z-potential greater than 30 mV [42,44], which suggests that the design of lipoplexes presents optimal characteristics to be a good carrier of siRNAs in CC therapy. The PDI shows the distribution of individual molecular masses in a sample, and a low PDI indicates a narrow particle size distribution [46]. Therefore, we can infer that the particle size distribution of the designed formulation is acceptable because the PDI does not exceed 0.300 ± 0.051. Several reports have shown that lipid concentrations and the preparation process of formulations are related to transfection efficiency and target gene silencing [37,47].

Accordingly, the system designed in this work was based on the use of DOTAP, a cationic lipid that allows binding to negatively charged molecules such as siRNA, cholesterol, a neutral lipid that can stabilize and stiffen lipid membranes, and DOPE, which enhances siRNA transfection by destabilizing the liposomal membrane in the cell cytoplasm [48]. Likewise, the physicochemical characteristics of the lipoplexes are important for efficient transfection. For example, an appropriate particle size allows lipoplexes to avoid activating the immune response and prevents rapid elimination from the circulatory system. For this reason, the system also contained DSPE-PEG2000, a polymer that extends the lifetime of liposomes in circulation and is nonimmunogenic due to its neutral nature; in addition, it protects siRNA against degradation [49]. It is important to highlight that the Z-potential is a parameter that determines the stability of the particle, considering that a value > +30 mV can minimize the aggregation of the liposomal particles [50]. Our results showed a Z-potential greater than 30 mV, demonstrating that the formulated product exhibited the optimal characteristics of a good delivery system. Based on the above, the next step was to verify the stability of the siRNA coupled to the nanosystem.

### 3.2. Ensuring the Stability of Lipoplexes-HPV16 E6 Is Crucial for Their Successful Application as siRNA Delivery Systems

The close relationship between the E6 and E7 oncoproteins of HPV16 and their interactions with various molecular targets are related to the dysregulation of multiple cellular mechanisms, which contribute to the onset and progression of CC. Due to the pivotal roles of the E6 and E7 oncoproteins in CC, interest in developing therapeutic strategies to target and inhibit these oncoproteins is growing. These interventions aim to interrupt the processes associated with carcinogenesis. Although several treatments for CC are available, many of these treatments are invasive or cytotoxic and have limited bioavailability. Therefore, despite the significant relevance of liposome design, our focus in this research was in the development of a precise and highly effective siRNA capable of suppressing the expression of the E6 oncoprotein of HPV 16 while simultaneously ensuring minimal cytotoxicity. The importance of siRNAs lies in their ability to target and silence specific genes, as they can modulate gene expression with high precision; however, specific characteristics of the designed sequence are required to inhibit the expression of the target sequence (mRNA) ensuring its binding [51], such as thermodynamic characteristics, the G/C content (around 50%), a 5-end rich in A/U, not being homologous with other genes, and the absence of secondary structure formation, to name a few [52,53].

**Figure 1:**
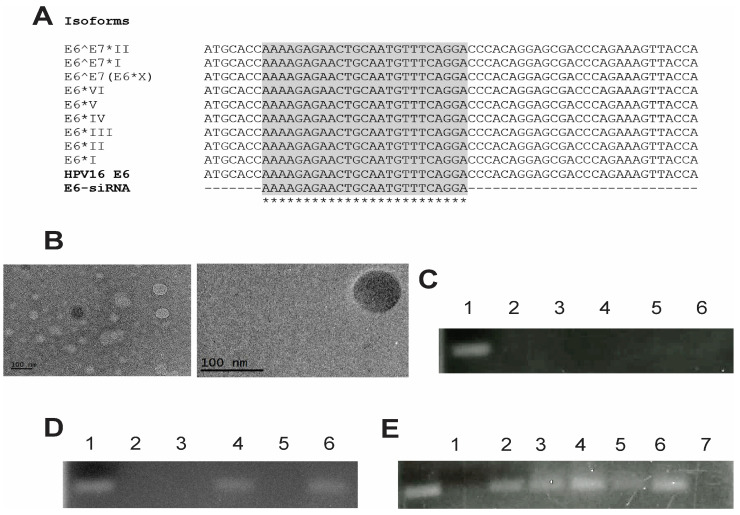
Design and evaluation of siRNAs. (**A**) Alignment between complete E6 sequences of VP16 and its main isoforms, aligned with the E6 siRNA. (**B**) Transmission electron microscopy (TEM), visualization of lipoplexes-PEG-HPV16 E6. (**C**) A 4.5% agarose gel was used to evaluate siRNAs’ binding to liposomes and liposomes-PEG. Lane 1, nonencapsulated control siRNA; lane 2, liposome; lane 3, lipoplexes-HPV16 E6; lane 4, liposomes-PEG; lane 5, lipoplexes-PEG-HPV16 E6. (**D**) Triton X-100 assay. Lane 1, uncapped control siRNA; lane 2, lipoplexes-HPV16 E6 without Triton X-100; lane 3, lipoplexes-HPV16 E6 with Triton X-100; lane 4, lipoplexes-HPV16 E6 without Triton X-100; lane 5, lipoplexes-PEG-HPV16 E6 with Triton X-100. (**E**) RNase assay. lane 1, control siRNA; lane 2, control siRNA with RNase; lane 3, control siRNA with RNase A/RNase OUT; lane 4, lipoplexes-HPV16 E6 with RNase A/RNase OUT (Triton X-100-treated); lane 5, lipoplexes-HPV16 E6 treated only with Triton X-100; lane 6, lipoplexes-PEG-HPV16 E6 with RNase A/RNase OUT (Triton X-100-treated); and lane 7, lipoplexes-PEG-HPV16 E6 treated with Triton X-100 alone. Each assay was performed in triplicate. * Represents conserved sequence (identical).

The previously mentioned characteristics were considered for the design of our siRNA. An essential and fundamental element in the design of this siRNA was to ensure it met the condition of identifying sequences of E6 isoforms expressed by the CaSki cell line, such as E6*I, E6*II, as well as the full E6 sequence [34]. However, to obtain a more effective siRNA, its design was based on the sequences of the E6 isoforms described in the literature [54,55,56,57,58,59], as can be observed in Figure 1A. In this design, we obtained a sequence capable of coupling in silico analysis to the sequences of at least 10 isoforms, suggesting it could be a good candidate for CC therapies.

To support these results and visualize the morphology of the coupling between siRNA and liposome, Figure 1B shows the spherical morphology of the lipoplexes-PEG-HPV16 E6 observed by transmission electron microscopy.

**Figure 2:**
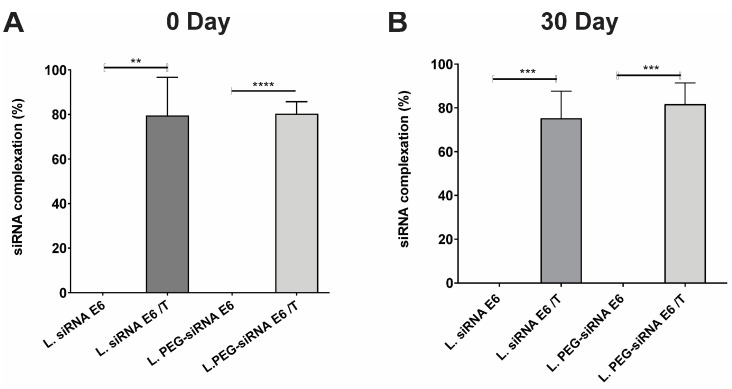
The siRNA binding efficiency. The siRNA binding in the different formulations was quantified using the Quant-iTTM RiboGreen^®^ RNA Assay Kit. The percentages of siRNA binding on day 0 and after 30 days are shown in (**A**,**B**), respectively. The formulations were treated with or without Triton X-100 (10%), and the results were calculated as encapsulated siRNA/total siRNA × 100. Statistical analysis was performed using ANOVA followed by Dunnett’s multiple comparison test (** *p* < 0.005, *** *p* < 0.0005, and **** *p* < 0.0001).

Once the siRNA with all the required characteristics was obtained and coupled to the liposomes, a delayed electrophoresis assay was performed to evaluate the efficiency of siRNA binding to the liposomes. This assay allowed us to visualize and analyze the binding interactions between siRNA molecules and liposomes, providing information on complex formation. The successful binding of siRNA to both pegylated and non-pegylated lipoplexes-HPV16 E6 can be observed in Figure 1C. The absence of any visible band in the gel indicated that the siRNA was effectively bound to the cationic lipids of the liposomes, specifically DOTAP. To demonstrate that the siRNAs were coupled to the liposomes, liposomes-PEG (lipoplexes-PEG-HPV16 E6) were digested with Triton X-100 at 10X and the siRNAs coupled to the liposomes were visualized (Figure 1D). These results indicated that the siRNAs coupled to the liposomes (lipoplexes-HPV16 E6) and that only the destabilization of the liposome membrane would allow siRNA uncoupling. In addition, the protective effect of the liposomes was evaluated in the presence of RNases (Figure 1E). This clearly showed that both liposomes and PEG-liposomes effectively protected E6 siRNA from degradation caused by RNases. This protective effect is highly significant, as it indicates that, in in vitro assays and potentially in in vivo settings, the integrity and functionality of the siRNA can be retained for an extended period. To confirm the presence of siRNA in the PEG-HPV16 E6 lipoplexes, we quantified the amount of siRNA using the RiboGreen^®^ assay. As shown in Figure 2A,B, the percentage of siRNA bound to lipoplexes and PEG-lipoplexes at 0 and 30 days was evaluated. The results showed that the percentages of siRNA bound to liposomes and liposome-PEG were 79.55% and 80.34%, respectively (Figure 2A), demonstrating that the liposomes possessed high docking efficiency.

**Figure 3:**
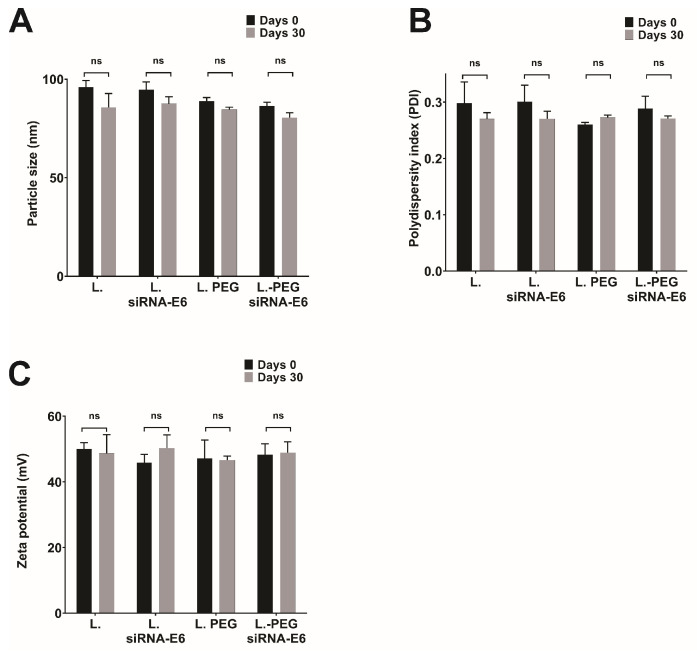
Stability evaluation of liposomes and lipoplexes. Comparative characterization of liposomes and lipoplexes before and after 30 days of storage at 4 °C. (**A**) Particle size (nm), (**B**) polydispersity index (PDI), and (**C**) zeta potential (mV). Each assay was performed in triplicate. (ns = not significant).

Stability tests were carried out after 30 days of storage at 4 °C. Figure 3 compares the particle size (Figure 3A), PDI (Figure 3B), and Z-potential (Figure 3C) before and after storage, demonstrating that the elapsed storage time of the lipoplexes-PEG-HPV16 E6 caused no significant differences in size, PDI, and Z-potential. Similarly, a retardation assay was performed to evaluate whether the siRNAs remained coupled to the lipoplexes-HPV16 E6 or lipoplexes-PEG-HPV16 E6 after 30 days, and the results showed the storage time did not alter the binding efficiency of the siRNAs. Additionally, after 30 days of storage, the percentage of siRNA coupled to lipoplexes-HPV16 E6 and lipoplexes-PEG-HPV16 E6 was 75.28% and 81.75%, respectively (Figure 2B). These findings complement previously obtained results confirming the stability and integrity of siRNA when coupled to lipoplexes-HPV16 E6, thus demonstrating that the storage period did not influence the stability or binding of siRNA to lipoplexes-HPV16 E6. These data showed that the size, Z-potential, and PDI were not affected by time. In addition, the high coupling percentages indicated that the siRNA remained bound to the liposomal formulations even after storage, suggesting that the electrostatic interactions between the siRNA and DOTAP, the cationic lipid used, remained intact for at least 30 days. These data agree with the observations reported by Hattori in 2020, which demonstrated that DOTAP-based pegylated liposomes and lipoplexes allow complete siRNA binding [42]. In addition, our system shares the characteristics of the zeta potential and PDI reported by Hattori with 1–3% PEG; however, in our formulation with 5% PEG, the particle size was within the same size range for liposomes and lipoplexes, which is an advantage of our system in stabilizing the particle size.

It has been demonstrated that DOTAP-based liposomes do not release siRNA during storage at 4 °C; the coupling efficiency remains stable at almost 95% [44]. On the other hand, Fisher’s results showed that the encapsulation efficiency of cationic liposomes based on DOTAP with 10% PEG was 60.03% [60]. Likewise, when the physicochemical characteristics of liposomes and lipoplexes-PEG-HPV16 E6 were evaluated after 30 days of storage, the values were similar to those obtained on day 0, which suggests that a safe, stable, and effective system for in vitro administration was established. These results are similar to those obtained by Haghiralsadat, who showed that the physicochemical characteristics and the percentage of coupled siRNA remained stable after 6 months of storage at 4 °C, demonstrating that the presence of DSPE-mPEG_2000_ in the formulations allows the long-term maintenance of stability [61]. Thus, the addition of this polymer to our formulation allowed the zeta potential to remain stable for 30 days. In summary, the data demonstrate that both HPV16 E6 lipoplexes (with and without PEG) exhibit excellent physicochemical stability and high siRNA binding capacity.

### 3.3. Silencing of E6 Oncoprotein Expression in CaSki Cells

Considering this, our focus was on developing an siRNA that met the required characteristics of a good siRNA, aiming to silence the expression of the oncoprotein E6 of HPV 16 in vitro assays. Since it has been documented that only a limited fraction of designed siRNAs are functional, this dependence lies in their sequence, as the silencing effect of an siRNA will be efficient as long as it demonstrates complete complementarity to the target gene sequence [62].

**Figure 4:**
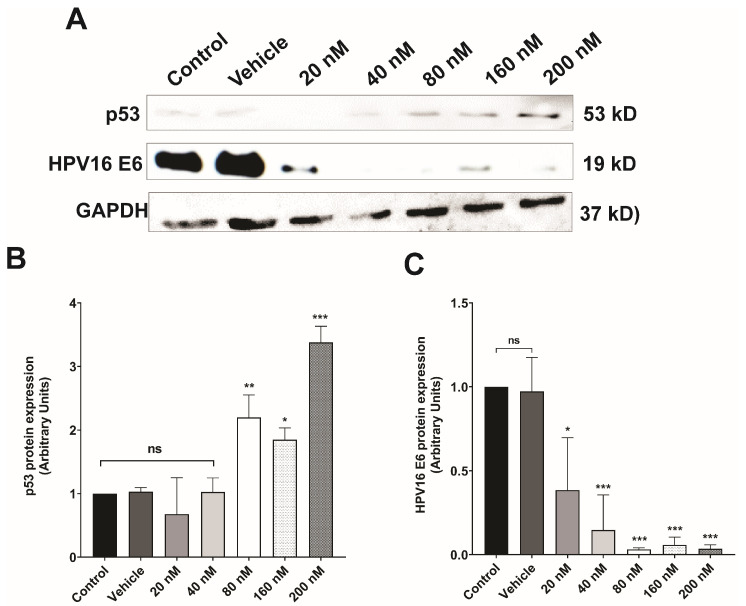
The protein expression levels of E6 and p53 in CaSki cells treated with lipoplexes-PEG-HPV16 E6 were evaluated. (**A**) A representative Western blot image, depicting the detection of the E6 and p53 proteins. GAPDH was used as a loading control. (**B**) Densitometric analysis of p53 expression. (**C**) Densitometric analysis of E6 expression. Statistical analysis was performed using ANOVA followed by Dunnett’s multiple comparison test (* *p*< 0.05, ** *p* < 0.005, and *** *p* < 0.0005). The assay was performed in triplicate (*n* = 3), (ns = not significant).

Dose–response assays were used to evaluate the effect of lipoplexes-PEG-HPV16 E6 on HPV16 E6 oncoprotein expression after 48 h of treatment with different concentrations of siRNA (20, 40, 80, 160, or 200 nM) (Figure 4A). Densitometric analysis revealed that treatment at a concentration of 20 nM significantly decreased E6 oncoprotein expression; however, concentrations of 80, 160, and 200 nM inhibited E6 expression the most effectively (Figure 4B). In addition, the expression of p53, a target protein of HPV 16 E6, was evaluated. The results showed that treatment with 80 nM siRNA restored p53 expression (Figure 4C) to the basal level observed in CaSki cells (control and vehicle). These results demonstrated the efficacy of the system since increasing siRNA concentrations (80, 160, and 200 nM) both decreased E6 oncoprotein expression and restored p53 expression in our study model, demonstrating that not only the expression of E6 oncoprotein but also its function was reduced (Figure 4C).

E6 is a major oncoprotein that interacts with various molecular targets that mediate cellular processes such as apoptosis, transcription, chromosomal stability, differentiation, and proliferation [63,64]. This is why silencing E6 through siRNA is highly advantageous and only affects positive HPV-infected cells. However, the transfection of siRNAs in in vitro models is limited by their easy degradation by exonucleases and the negative charge of the cell membrane, which leads to the easy formation of complexes with positively charged molecules [42]. The development of liposomes has allowed us to evaluate the effect of these siRNAs on cell lines by protecting them from degradation and complex formation.

The development of CC is closely associated with the degradation of the tumor suppressor protein p53, which is induced by the E6 oncoprotein expressed by high-risk human papillomaviruses (HPVs). This degradation mechanism disrupts key cellular processes, leading to the progression of CC [65]. In response to the critical role of cellular alterations in disease progression, therapeutic systems have been developed to inhibit or block target molecules involved in these processes. These innovative approaches are designed to restore cellular homeostasis and attenuate the detrimental effects of molecular dysregulation; accordingly, the development of small interfering RNAs (siRNAs) targeting the E6 oncoprotein has emerged as a promising strategy to effectively decrease E6 expression and restore the balance of p53 signaling pathways. In particular, the liposome-coupled delivery of siRNAs has shown great potential to increase the therapeutic efficacy of this approach [44]. The results of our dose–response assay showed that the use of 160 and 200 nM siRNAs coupled to liposomes (lipoplexes-PEG-HPV16 E6) significantly decreased the expression of E6 and increased the expression of p53 in CaSki cells. These findings show that after more than 30 days of storage, these formulations retain the ability to inhibit E6 expression in HPV-positive cells in vitro.

### 3.4. In Vitro Evaluation of the Effect of Lipoplexes-HPV16 E6 on Cell Viability

**Figure 5:**
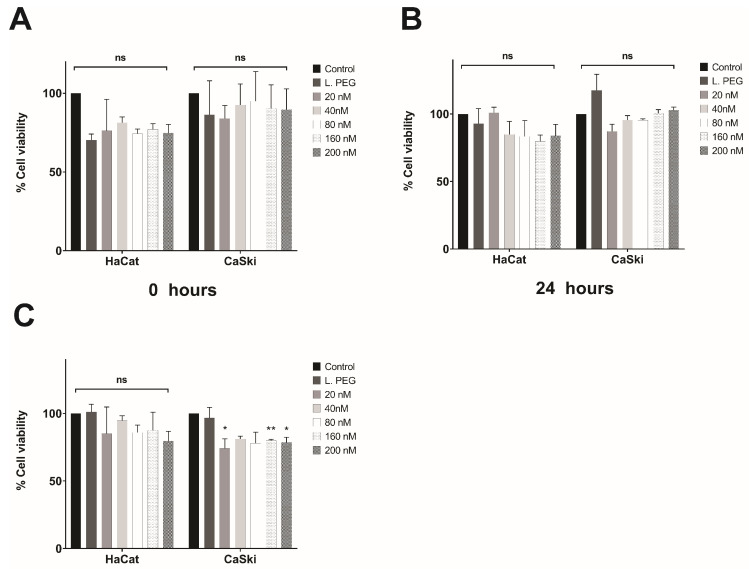
Cell viability. Effect of liposomes and lipoplexes-HPV16 E6 with PEG. HaCaT and CaSki cells were treated with lipoplexes-HPV16 E6 with PEG at 0 h (**A**), 24 h (**B**), and 48 h (**C**) at different molar concentrations. L.PEG was used as a cytotoxicity control; (* *p* < 0.05 and ** *p* < 0.005) (ns = not significant).

MTT assays were performed on the CaSki cell line (HPV16-positive), and the HaCaT cell line (HPV-negative) was used as a control. For dose–response assays, the cells were treated with different siRNA concentrations (20, 40, 80, 160, and 200 nM) and evaluated at different times (0, 24, and 48 h), as shown in Figure 5. In both the HaCaT and CaSki cell lines, the cytotoxic effect was independent of the tested concentration and duration, as no significant cytotoxic effects were observed (Figure 5A–C). These results collectively demonstrate that the efficacy and specificity of the lipoplex-PEG-HPV16 E6 treatment in reducing cellular proliferation do not depend on concentration or exposure time. However, in the CaSki cell line, significant differences were observed between the controls and the tested concentrations at 48 h. This could be due to the silencing of the E6 oncoprotein, which may be causing a greater cytotoxic effect.

Once optimal lipoplexes were obtained that inhibited E6 oncoprotein expression and restored expression of p53 (a key regulator of cell proliferation), the inhibition of E6 function was evaluated by cell proliferation assays, which showed that the lipoplexes-PEG-HPV16 E6 were able to inhibit proliferation of the HPV16-positive cell line at 48 h after transfection. Other studies have shown that the use of specific siRNAs can significantly inhibit cell growth [44,66]. Likewise, the effect of lipoplexes-PEG-HPV16 E6 on an HPV16-negative cell line was evaluated, and the results revealed normal cell growth. This shows that E6 siRNA specifically affects the HPV16-positive CaSki cell line, which is consistent with previous reports in the literature.

### 3.5. Lipoplex Treatment Suppresses the Migration and Invasion of CaSki Cells

**Figure 6:**
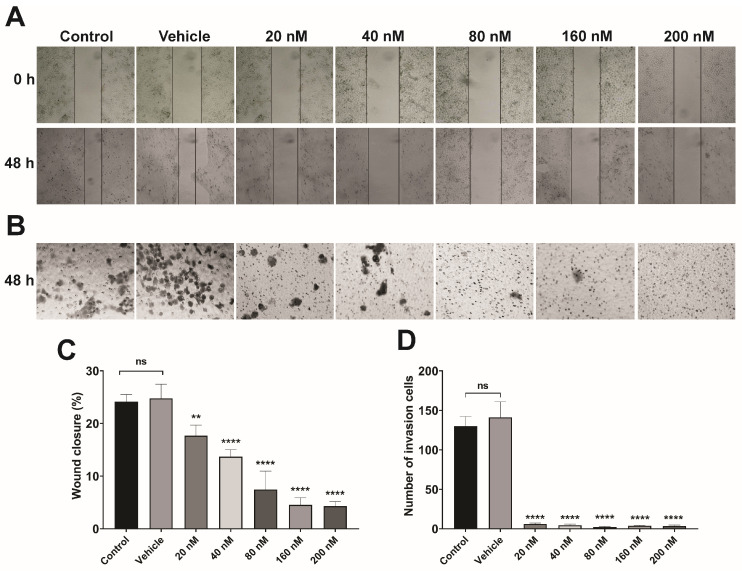
Inhibition of cell migration and invasion. (**A**) Representative images of the effects of lipoplexes-PEG-HPV16 E6 at different siRNA concentrations on migration by wound closure assay. (**B**) Representative images of the effects of lipoplexes-PEG-HPV16 E6 at different siRNA concentrations on invasion by Transwell chamber assay. Control (untreated cells) and vehicle (H_2_O). (**C**) Scratch wound closure assay in CaSki cell line. (**D**) Bar graphs represent the mean account of cell in five regions of each well at random (*n* = 5) invasion in Transwell assay in CaSki cell line. The CaSki line cells were treated with lipoplexes-HPV16 E6 with PEG at different nM concentrations. Statistical analysis was performed using ANOVA followed by Dunnett’s multiple comparison test (** *p* < 0.005 and **** *p* < 0.0001). Each assay was performed in triplicate (ns = not significant).

Cell migration and invasion are two of the most critical events in the progression of cervical carcinogenesis and are key biological events for metastasis and dissemination to other organs [67]. To evaluate the effect of treatment on both cellular processes, Transwell chamber assays of wound closure and invasion were performed only on the CaSki cell line (HPV16-positive) because no cytotoxic effects were observed in the control cell line HaCaT (HPV-negative). The results showed a significant decrease in cell migration starting at 20 nM siRNA (** *p* < 0.005), with greater decreases observed at 40, 80, 160, and 200 nM (**** *p* < 0.0001) (Figure 6A) after 48 h of treatment. Interestingly, the cell invasion assay showed that lipoplexes-PEG-HPV16 E6 had greater treatment efficacy, achieving almost total inhibition compared to the controls at all applied concentrations (20, 40, 80, 160, and 200 nM) (**** *p* < 0.0001) (Figure 6B). The assays showed that the effect of treatment with lipoplexes-PEG-HPV16 E6 depends on the applied dose and the targeted process and that this treatment is an effective alternative at multiple stages of CC progression.

The use of siRNAs with molecular targets that are involved in biological processes related to HPV oncogenesis, such as cell migration and invasion, has been shown to significantly inhibit these processes in CC cell lines [68,69,70]. E6 regulates processes such as migration, proliferation, and invasion by suppressing genes involved in these processes. The siRNA-mediated degradation of this oncoprotein can restore the expression of these key genes. However, siRNA transfection has the disadvantage that siRNA is easily degraded, and the use of lipid systems coupled to siRNAs (lipoplexes) increases the inhibition of the target molecules.

**Figure 7:**
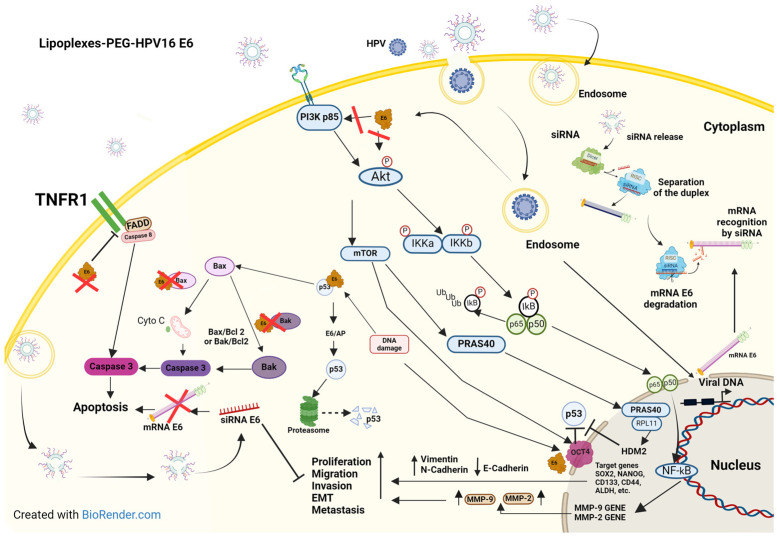
Proposed model of the interaction of lipoplexes-PEG-HPV16 E6 with target proteins related to proliferation, migration, apoptosis, and invasion. Treatment with E6 lipoplexes inhibited the interactions of the E6 oncoprotein with several of its target molecules, such as AKT, PI3K, and FADD, that are involved in various biological events of cervical carcinogenesis [71,72,73,74]. The arrows signify activation, the T-shaped lines signify inhibition, and the dashed arrow signifies degradation.

We hypothesized that the mechanisms of action of lipoplexes in our study model could be explained by the activation of various signaling pathways, as described by other research groups and illustrated in Figure 7 [71,72,73,74]. Treatment with lipoplexes-PEG-HPV16 E6 proved to be an efficient alternative therapy for inhibiting E6 oncoprotein-mediated processes in CaSki cells. The first step is the internalization of lipoplexes-PEG-HPV16 E6 into cells; then, the E6 siRNAs are released and directed toward the E6 mRNA for degradation through different processes. HPV integration induces E6 expression, which is related to the activation of various pathways that are involved in CC [75]. The cell death process mediated by the E6 oncoprotein is regulated at a minimum of three important points: In the first one, the direct interaction of the E6 oncoprotein with the tumor necrosis factor (TNF) receptor promotes inactivation by blocking the intracellular death domain that interacts with the Fas-associated death domain (FADD) (a direct regulator of the caspase pathway), thereby inhibiting cell apoptosis [71]. FADD is involved in several apoptotic pathways, and studies have shown that in HPV-positive cells, the presence of the E6 oncoprotein prevents apoptosis and allows cell survival [76]. The next checkpoint is the degradation of the canonical target of E6, the p53 protein; p53 is inhibited by the ubiquitin ligase E6-AP [E6-associated protein (E6/AP)] recruitment, which induces p53 degradation via the proteasome pathway and thereby blocks cell cycle arrest and apoptosis [77]. Finally, the third checkpoint is mediated by the phosphatidylinositol 3-kinase (PI3K) pathway, which, when activated directly by E6, acts as a negative regulator of apoptosis through the phosphorylation of PRAS40/mTOR, leading to an increase in cell proliferation [72,78], as illustrated in Figure 7. The activation of the PI3K/AKT pathway by the oncoprotein E6 is also involved in the increased secretion of MMPs through the downstream activation of nuclear factor kappa B (NF-κB), which in turn promotes cell migration and invasion [79]. Moreover, Akt activation can induce octamer-binding protein 4 (OCT4) phosphorylation, which is related to the increased tumorigenicity and self-renewal capacity of CSCs through the transcriptional regulation of OCT4 target genes [Transcription factor SOX-2 (SOX2), Homeobox protein NANOG (NANOG), CD133 isoform H (CD133), CD44 antigen (CD44), aldehyde dehydrogenase (ALDH), etc.] [73,74]. OCT4 has been proposed as a key transcription factor in the progression of CC since it has been observed that in patients and cell lines (HPV-positive) derived from patients with more advanced stages (stages III and IV) of CC, this protein is overexpressed [73,74]. OCT4 orchestrates EMT (a key process in cervical metastasis) through the positive and negative regulation of genes involved in various molecular events (migration, secretion of MMPs, and cell invasion) [80,81]. According to the results obtained in this study, lipoplexes-PEG-HPV16 E6 could be a reliable carrier for the delivery of new and highly specific gene therapies.

Furthermore, treatment with the formulation developed herein effectively inhibited three processes mediated by the E6 oncoprotein, particularly cell invasion. Finally, pegylated DOTAP-based lipoplexes coupled to E6 siRNAs proved to be highly efficient delivery systems for inhibiting E6 oncoprotein expression and its functions in biological processes related to CC development.

## 4. Conclusions

In this study, pegylated liposomes coupled to E6 siRNAs were developed. This liposomal formulation showed a high rate of siRNA coupling, and the size, PDI, Z-potential, and siRNA coupling to the liposomes remained stable after 30 days of storage. In in vitro assays, the use of pegylated lipoplexes-PEG-HPV16 E6 efficiently silenced E6 expression and restored p53 expression in CaSki cells. Additionally, functional assays demonstrated that the degradation of the E6 oncoprotein induced by lipoplexes-PEG-HPV16 E6 decreased proliferation, migration, and invasion in our study model. Our findings suggest an alternative method to improve the delivery of conventional therapies in patients with CC. Future studies should be developed to elucidate the signaling pathways involved in the regulatory effects of lipoplexes on E6 oncoprotein-mediated cellular processes, which will provide a more specific understanding of the mechanisms underlying these effects.

## Figures and Tables

**Table 1:** Physicochemical characteristics of lipoplexes-HPV16 E6 with and without PEG: particle size, PDI and Z-potential.

DOTAP:DOPE:Chol (2:1:1)	%PEG	Particle Size (nm)	PDI	Z-Potential (mV)
Liposome	0	95.93 ± 5.89	0.298 ± 0.065	50.03 ± 3.33
Liposome + siRNA E6 (lipoplexes)	0	94.66 ± 6.87	0.300 ± 0.051	45.9 ± 4.33
Liposome-PEG	5	88.96 ± 2.98	0.264 ± 0	47.16 ± 9.73
Liposome-PEG + siRNA E6 (lipoplexes)	5	86.42 ± 3.19	0.288 ± 0.038	48.3 ± 5.72

PDI: polydispersion index. Each value is represented as mean ± SD of three measurements per sample.

## Data Availability

Data are contained within the article.
